# Prevalence of alcohol and drug dependence in rural and slum population of Chandigarh: A community survey

**DOI:** 10.4103/0019-5545.31517

**Published:** 2007

**Authors:** B. S. Chavan, Priti Arun, Rachna Bhargava, Gurvinder Pal Singh

**Affiliations:** Department of Psychiatry, Govt. Medical College and Hospital, Sector - 32, Chandigarh, India

**Keywords:** Drug dependence, alcohol dependence, community survey

## Abstract

The present epidemiological survey was conducted by the department of psychiatry, Govt. Medical College and Hospital, Chandigarh to estimate the pattern of alcohol and other substance dependence in rural and slum dwellers population of Chandigarh. In this survey 6.88% individuals of the total population surveyed (2992) fulfilled dependence criteria of ICD-10. Alcohol was the primary substance of dependence for majority of urban slum substance users and rural areas users. Age at first drug use was 20.89 ± 5.31 years (mean ± S.D) among rural population and 19.75 ± 5.4 years (mean ± SD) in urban slums. Majority of them reported having health related complications (85.71%) followed by family problems (77.31%) due to drug dependence. This survey reflects the need to intensify efforts at the community level to reach the unreached.

## INTRODUCTION

Drug dependence has been showing a rising trend all over the world including India, perhaps as a result of newer and greater stresses related to rapid changes in life styles. Drug dependence is a growing problem and consequences of drug dependence cost heavily to the community and form a major health problem.[1] Alcohol and drug related behavioural and medical complications are a major concern for policy planners and health professionals of most of the countries. This problem has received some attention in the recent years among the general public and mental health professionals. In last three decades, many epidemiological surveys have been carried out in India to assess the prevalence of alcohol and drug users.

Elnager *et al*[2] reported a prevalence rate of 13 per 1000 in West Bengal, while Nandi *et al*[3] gave a figure of 0.94 per 1000 of the total population for the same state. Similarly in Uttar Pradesh, Dube and Handa[4] reported that 22.8 per 1000 were dependent on alcohol and drugs while Thacore[5] from Lucknow gave a figure of 18.55 per 1000. Important finding of these studies is that alcohol was the commonest substance used (60-98%) followed by cannabis use (4-20%). Epidemiological surveys also revealed that 20-40% of subjects above 15 years are current users of alcohol and 10% of them are regular or excessive users.[6–8] In a rural population of Uttar Pradesh alcohol was found to be the commonest substance abused (82.5%) followed by cannabis (16.1%).[9] Deb and Jindal[10] in a survey of 4 villages in Punjab found that 78.28% of the population used alcohol whereas in the same state Lal and Singh[7] reported it to be 9.13% of total population surveyed. Varma *et al*[11] found that rates of current use of alcohol in Punjab were 45.9% in Jalandhar and 27.7% in Chandigarh whereas it was 28.1% in rural areas of Punjab.[12] Shukla[13] reported that 38.3% of the rural population in Uttar Pradesh was habitual users. In a study conducted in rural community in Bihar prevalence of alcohol/drug use was found to be 28.8% of the study population.[14] Meena *et al*[15] in an urban population in Rohtak district of Haryana revealed a prevalence rate of 19.78% of the study population. In a meta-analysis of 13 psychiatric epidemiological studies it was found that the prevalence rate of alcohol/drug use was 6.9 per 1000 population.[16]

A National household survey was conducted in India for estimating the extent of substance dependence for alcohol and opiates. The data was collected between March 2000 and November 2001. The diagnosis of dependence was arrived using ICD-10 criteria. In this study, the current prevalence of alcohol was 21.4%, cannabis 3.0%, Heroin 0.2%, opium 0.4% and other opiates 0.1%. Another important finding of this survey was that in the range of 17-29% of current users of various substances was dependent users.[17]

In majority of these epidemiological surveys complications associated with alcohol and drug abuse have not been addressed. Also, use of other psychoactive substances (tranquilizers and hypnotics) was not studied in most surveys. More so, in Chandigarh not much work has been done in rural and slum areas, hence there was a need for assessment of prevalence of drug and alcohol dependence in these areas. There is a very little information about pattern of drug dependence among Chandigarh population. The whatever information is available is outdated as a lot of changes have occurred in the last two decades in terms of emergence of newer substances and newer routes of consumption and previous research was carried out mainly on urban population of Chandigarh. The findings of these studies may not be applicable to the present scene. The results of urban population can not be generalized to the rural and slum population. Thus it was worthwhile to study the pattern of drug and alcohol use and its adverse effects on rural and slum colony population of UT, Chandigarh.

### Aims and objectives

This survey was undertaken with the following objectives:

- To study the sociodemographic characteristics of the individuals using alcohol and drugs in rural and slum areas of Chandigarh.
- To assess the pattern of alcohol and drug dependence.
- To study the adverse effect of alcohol and drug use on personal, health, family, occupation and social areas.

## MATERIALS AND METHODS

### Sample size and sampling method

In the 1991 census of Chandigarh the total population was 6,42,015 individuals, out of which the urban population comprising Chandigarh city, Manimajra town, Burail, Attawa, Buterla and Badheri was 5,75,829 persons. The balance rural population of 66,185 person was spread over 18 villages. Out of this rural and urban slum population 59,470 individuals were selected randomly for this survey in 5 villages (Khuda Alisher, Mani Majra (rural), Hallo Majra, Palsaura and Dadu Majra) and 5 slum area colonies (Labour colony Palsaura, Gandhi Madrasi and Sansi Labour Colony, Gawala Colony, Janta Colony, Sector 25) of Chandigarh. In the selected villages and slum areas, a fresh listing of household number was done because most of the household in these areas did not have a proper house number. From these households every 10^th^ house was included in this survey. The technique employed for sample selection was of stratified random sampling. In the selected houses, all residents 15 years and above were included in the study. Total 3000 individuals above 15 years of age (1500 Individuals from rural areas and 1500 from slum area colonies) formed the sample of this survey.

### Inclusion criteria

All individual above 15 years of age staying in the above mentioned area.

### Exclusion criteria

Individuals who were staying alone, suffering from major mental or physical disorder, mental retardation and stayed in Chandigarh for less than 6 months were excluded from this study.

### Assessment

Following assessment instruments were used:

1. Interview with head/key informant of family: Information was recorded about total numbers of individuals in the family alongwith their age, sex, marital status, occupation, education. Information was also recorded on type of family, religion, caste, family income and the number of past and current alcohol and drug users.
2. Assessment of drug users: This instrument schedule was administered to drug users (as per the report of key informant) to record information on the age of initiation of drug use, age of regular use, reason of initiation and maintenance as well as pattern of drug use (quantity, duration of dependence, frequency and route). This instrument was developed by AIIMS, New Delhi and was used in National Household survey.[17]
3. ICD-10 criteria[18] for substance dependence: These were used for making the diagnosis.
4. Assessment of adverse effects: A semistructured proforma was constructed to collect information on adverse consequences secondary to alcohol and drug use. In this instrument items pertaining to health, occupation, legal, family and marital and social adverse effects of alcohol and drug use were included.

### Procedure

#### Phase 1

Two field research workers (graduates) were recruited and they were given one month training regarding the procedure to carry out the community survey. The training was personally supervised by the prinicipal investigator. Field research workers were trained in the vernacular language of the villages and slum areas surveyed. They were trained in carrying out an interview to keep the confidentiality of information, unbiased and non judgmental attitude and respect the individuals as they are. As a part of pilot work, the investigators accompanied the field research workers for ensuring quality and to provide on job training in deficient areas. The families of the selected villages and slum areas were visited in this phase of the community survey. Panchayat members and community leaders were contacted and their approval was taken. The head of the family or key informant was contacted in the respective household. Head of family in large number of cases had knowledge about problem users. Individuals having problems with alcohol and drug use were identified. Informed consent was taken from each individual before conducting the interview. Initially a pilot study of two month duration on 50 individuals was conducted and results of the pilot study were reviewed and the methodology was refined.

#### Phase 2

After the pilot phase, the trained research staff personally contacted the identified persons and information from them was collected using assessment instruments number 2 to 4. The respondents were reassured of confidentiality of the response. This data collection was done with the active supervision by the authors of this study. Random checking was done regularly to ensure the reliability. The total duration of the data collection in the survey was 13 months.

The data were analyzed using frequency distribution to ascertain the proportion of those who were alcohol and drug dependent. Central tendency measure used was mean and variability measure as standard deviation was calculated.

## RESULTS

Total 2992 individuals were included in the data analysis. Majority of the individuals were in the age group of 15-24 years (38.27%). Males (54.4%) outnumbered females (45.6%) [Table 1]. Majority of the sample was illiterate (37.67%), retired from service and housewives (37.80%), Hindu (67.80%), married (73.80%) and came from joint and extended Families (54.18%). The sample was equally distributed among rural and slum areas. As per information given by the head of the family, 56 subjects from rural area and 182 from urban slums reported problems related to alcohol and drug use. These subjects were assessed in detail on proforma for alcohol and drug use. Out of these 47 and 159 individuals respectively fulfilled dependence criteria on ICD-10, which constituted 6.88% of total population [Table 2]. In urban slums, 10.7% of population was dependent while in rural area only 3.12% fulfilled dependence criteria. Alcohol was the primary substance of dependence for majority of both urban slum substance users (93.08%) and rural substance users (91.5%). Cannabis and nicotine dependence were more common in urban slums. Multiple drug dependence was found in 1 person from rural area and 5 from urban slums. Table 3 shows mean age at first use and mean age of onset of regular use, which is similar in both groups. Alcohol and drugs affected almost all areas of life including health (85.71%), family (77.31%), marital (70.59%) and occupational (64.28%) as shown in Table 4.

**Table 1:** Sociodemographic characteristics

Variable		Frequency	Percentage
Age (in years)	15-24	1145	38.27
	25-44	1329	44.42
	45-64	396	13.23
	>65	122	4.07
Sex	Male	1628	54.4
	Female	1364	45.6
Education	Illiterate	1127	37.67
	Middle	1001	33.40
	Inter	742	24.80
	Post graduate	116	3.88
	Not Known	6	0.20
Occupation	Employee	786	26.27
	Unskilled/semiskilled	591	19.75
	worker		
	Housewives/retired	1131	37.8
	Unemployed	466	15.58
	Not Known	18	0.60
Religion	Hindu	2031	67.88
	Sikh	756	25.27
	Others	205	6.85
Family structure	Nuclear	1371	45.8
	Joint/extended	1621	54.18
Marital status	Single	690	23.06
	Married	2208	73.80
	Others	94	3.14
Residence	Rural	1506	50.3
	Slums	1486	49.7

**Table 2:** Pattern of substance dependence

	Rural N = 1506		Urban slums (N=1486)		
Substance	Primary drug	Secondary drug	Primary drug	Secondary drug
Alcohol	43 (2.85)	2 (0.13)	148 (9.96)	8 (0.54)
Opioid	3 (0.2)	2 (0.13)	3 (0.2)	2 (0.13)
Cannabis	1 (0.06)	3 (0.2)	6 (0.4)	11 (0.74)
Nicotine	0	4 (0.26)	2 (0.13)	56 (3.77)
Total dependent	47	11	159	77

Figures in parentheses are in percentage

**Table 3:** Age at first use and regular use

Age	Rural	Urban slums
Age at first use (years)		
Mean	20.89	19.75
S.D.	5.31	5.4
Age at regular use (years)		
Mean	26.65	24.12
S.D.	8.94	7.32

**Table 4:** Complications due to dependence

Complications	Rural (N=56) N (%)	Slum area (N=182) N (%)	Total (N=238) N (%)
Health	42 (75)	162 (89.01)	204 (85.71)
Family	35 (62.5)	149 (81.9)	184 (77.31)
Marital	33 (58.92)	135 (74.18)	168 (70.59)
Occupation	35 (62.5)	118 (64.84)	153 (64.28)
Social	17 (30.4)	74 (40.7)	91 (38.23)
Financial	5 (8.9)	31 (17.03)	36 (15.12)
Legal	4 (7.1)	11 (6.04)	15 (6.30)

## DISCUSSION

In the present community based survey, we have studied the pattern of alcohol and drug use in slum and rural population of Chandigarh. Chandigarh is the capital of two states (Haryana and Punjab) besides being an Union Territory. It has attracted a large number of people from different parts of India and gradually Chandigarh has become a cosmopolitan city. Chandigarh also has a large population of slum areas, both authorized and unauthorized. The survey was completed over a period of 13 months. In last 25 years, this is the only community-based survey on this population in Chandigarh. This survey has the largest and most representative sample of any previous effort to describe substance use among rural and urban slums population in north India. Out of 2992 subjects, 206 (6.88%) fulfilled ICD-10 criteria for substance dependence. These findings are comparable to other surveys done in our country. Meena *et al*[15] reported that 10.34% of the total population was dependent on alcohol. In contrast, Ghulam *et al*[19] reported a higher figure of 20.5% of substance dependence in the survey carried out on urban population of Madhya Pardesh. In another survey undertaken in Punjab, Lal and Singh[7] reported that 11% of the users were alcohol dependent. As depicted in Table 1, 54.4% of the sample was constituted by male and 45.6% by the females. In our survey, no female reported use of any substance. In earlier surveys conducted in different parts of India, similar findings had been documented by Lal and Singh.[7] Ghulam *et al*[19] highlighted that gender is an important factor in drug taking behavior. Females constituted 10.9% of the total sample of users in the survey undertaken by Ghulam *et al*.[18]

Absence of alcohol and drug dependence in females in rural and slum areas raises doubt about underreporting. Thus, this aspect needs to be taken care of in future studies. In previous community surveys, it was documented that the heavier drug use by female may be less likely. We recognize that a study of drug use by a female requires special case finding strategies. Strong community and social pressure, particularly in rural and slum areas might be responsible for underreporting. Mean age of first use was 20.89 years in rural areas and 19.75 years in slum areas. Simillarly mean age of regular use was 26.65 years in rural area and 24.12 years in slum area. These results are comparable to the findings as reported by Ghulam *et al*[18] and Lal and Singh.[7] This was an interesting finding of the present survey that in urban slum population both the age at first use (mean±S.D 19.75 yrs ± 5.4) and regular use (mean±S.D 24.12 yrs ±7.32) of alcohol and substance was earlier than in rural population. Alcohol was the most commonly used substance.

In our survey opium use was low in rural areas, which is in contrast to the findings of earlier surveys carried out in rural areas of Punjab.[7] Rural areas of Chandigarh are different from the villages of Punjab in terms of socio-cultural factors, educational status and civic amenities and stringent legal restrictions. High use of opium in rural areas of Punjab has been associated with farming sector. In Chandigarh, there is very little agricultural land and thus people are engaged in other occupations. Alcohol is cheap and easily available in Chandigarh. Other reason of low incidence of opium use in our study may be due to under reporting by family members. Field investigators observed that in few cases, spouse reported regular drug use while mother or father denied it.

This survey endorses the earlier findings that alcohol and drug affect almost all areas of life including occupation, health, family, marital, social and finance. Large proportion of substance users reported problems relating to physical health (86%), family (77%), marital (70%) and occupational (65%). Financial problems were reported by 15.12% of individuals in this survey. In this survey, there was a difference in profile of complication in two different study populations. In urban slum, substance users have more complication due to substance dependence. 89.01% of slum dependent persons had health related complication which was more than rural population (75%). Similarly family related complications (81.9%), marital problems (74.18%) and social and financial complications were more prevalent in urban slum subjects as compared to rural population.

Many of the substance users are not motivated toward help seeking and hence require treatment delivery at their door steps. This survey differs in some aspects from earlier community surveys done in India. In addition to information about the extent and nature of substances used, it also narrated information regarding adverse complication because of psychoactive substances. The overall findings of this survey do suggest the need for comprehensive treatment package including medical assessment and treatment of associated physical health problems as well as intervention in associated social issues related to substance use.

Some limitation of this survey includes use of a semistuctured assessment instrument and findings can't be generalized to the whole area of Chandigarh. Sample was restricted to rural and slum area population and inability to use better statistical measures due to sample characteristics.
